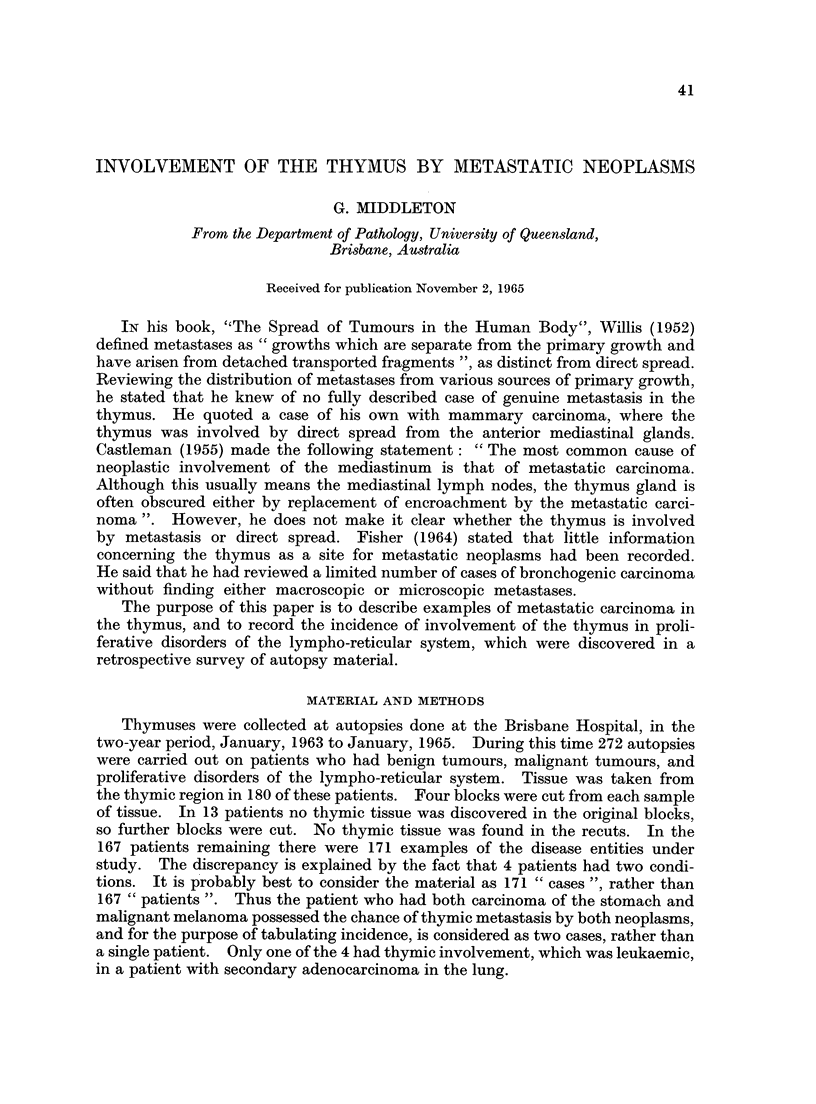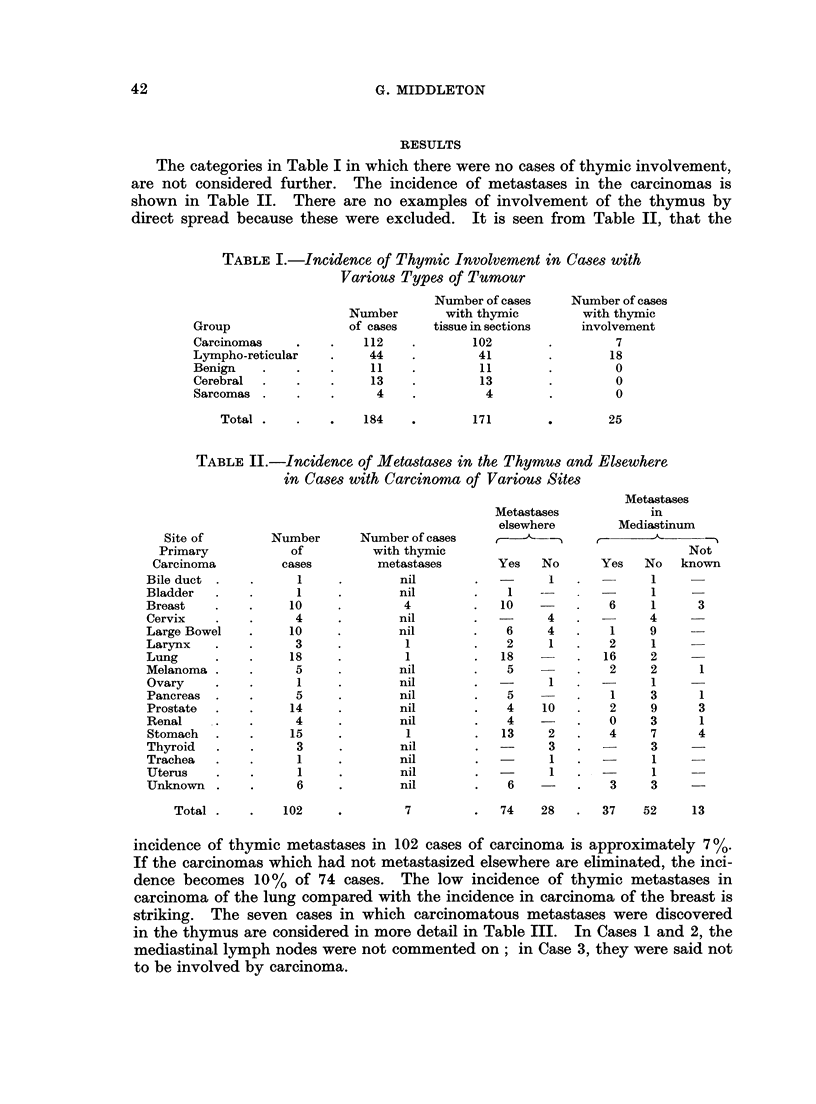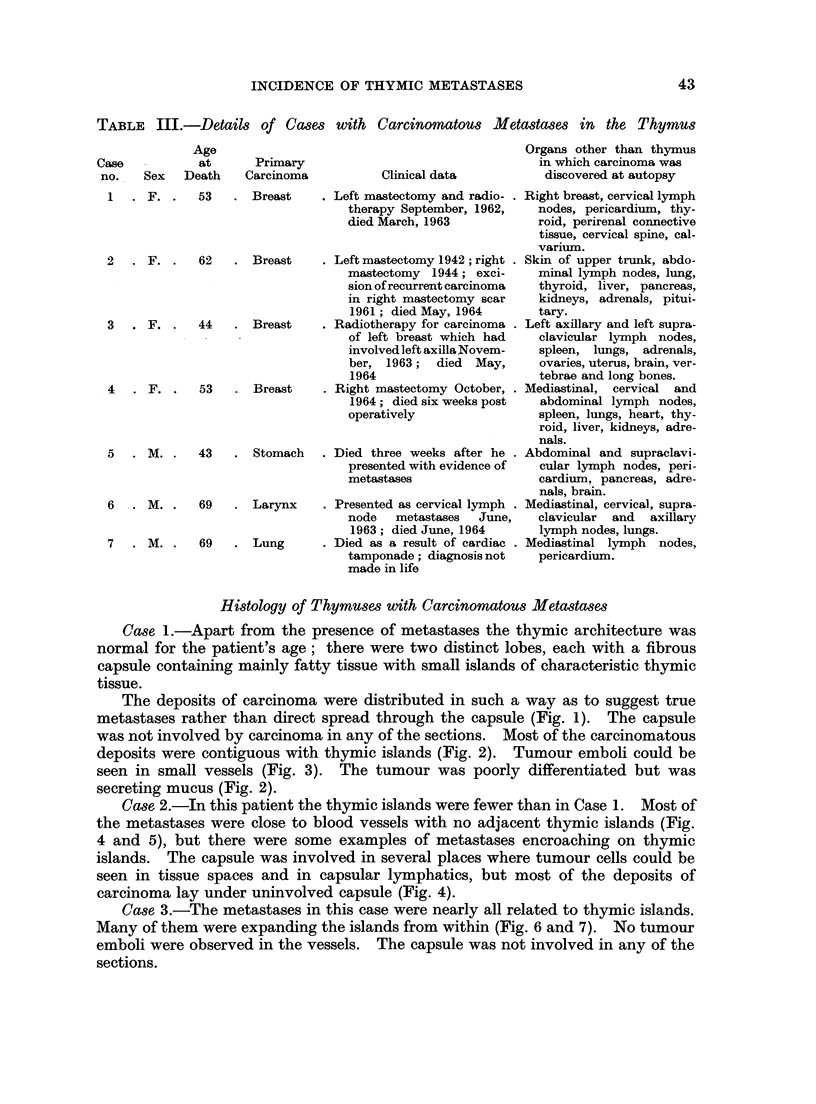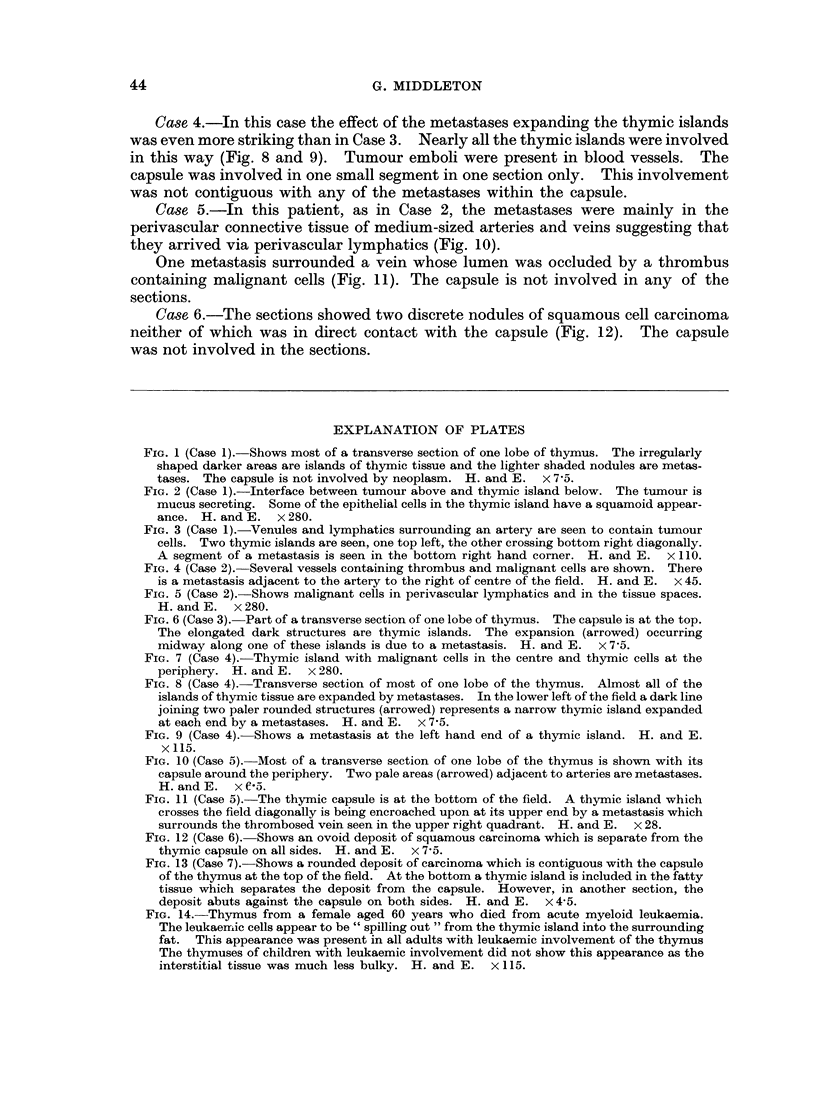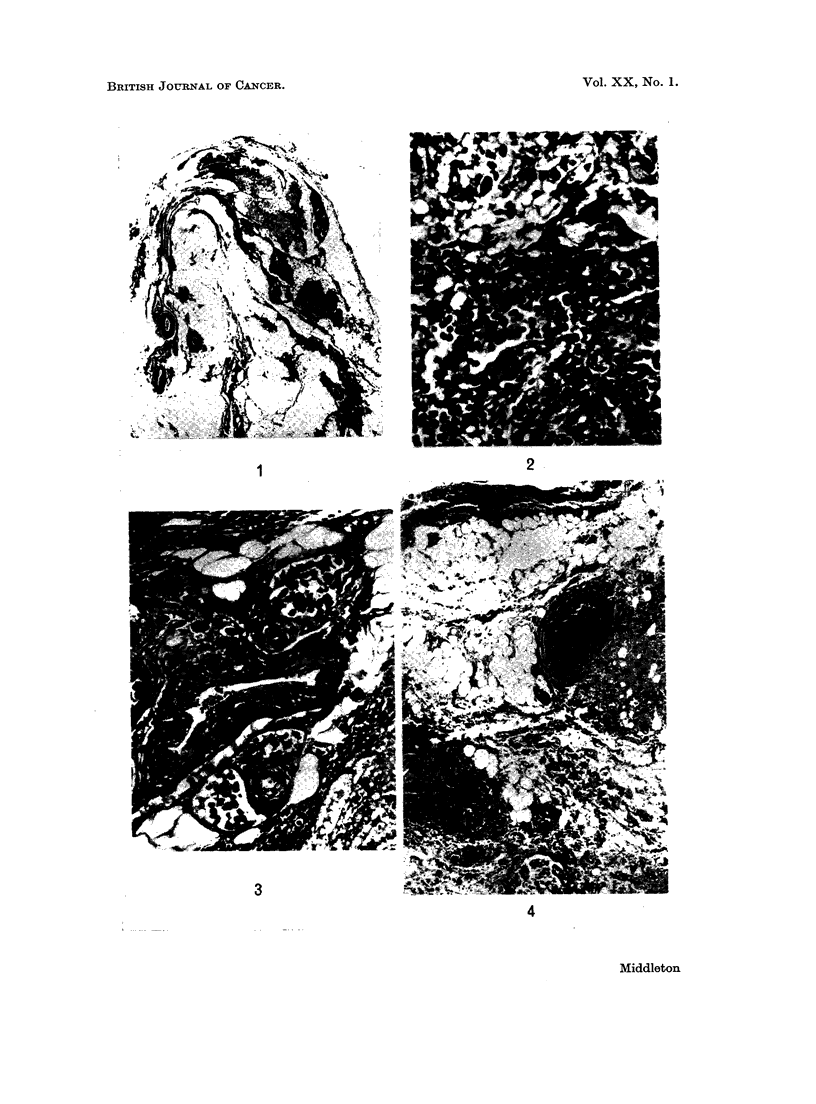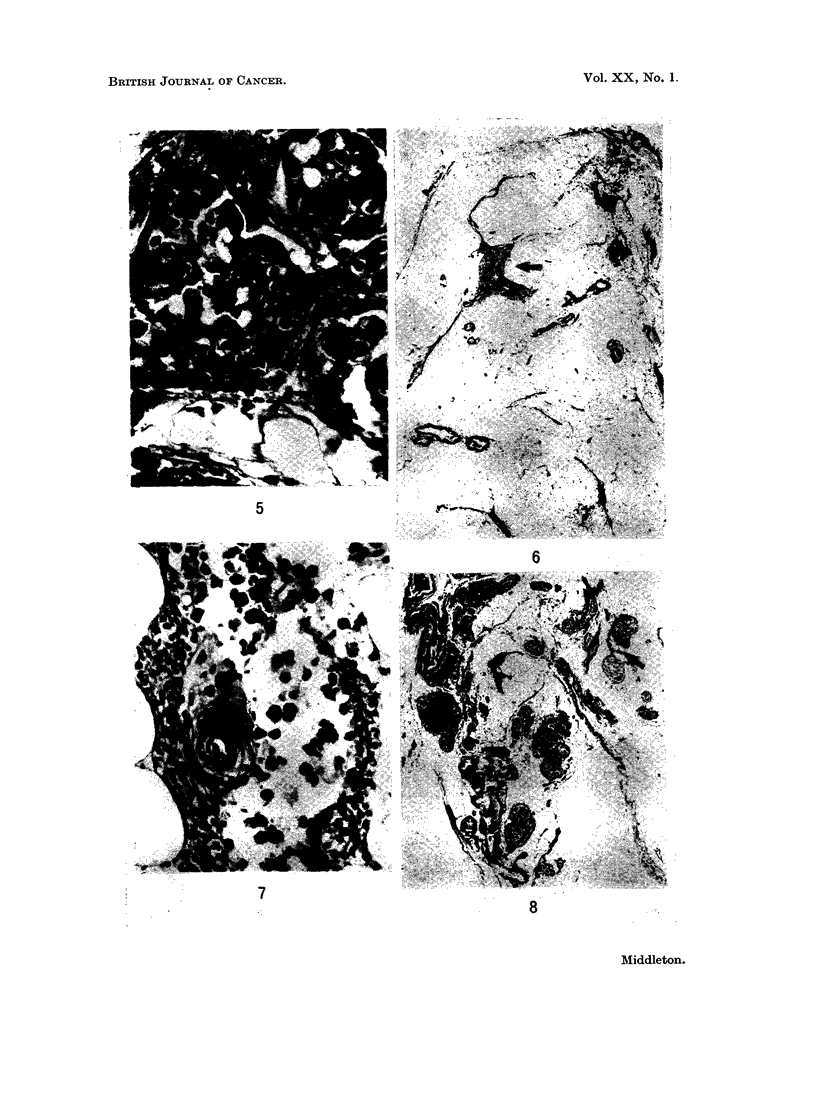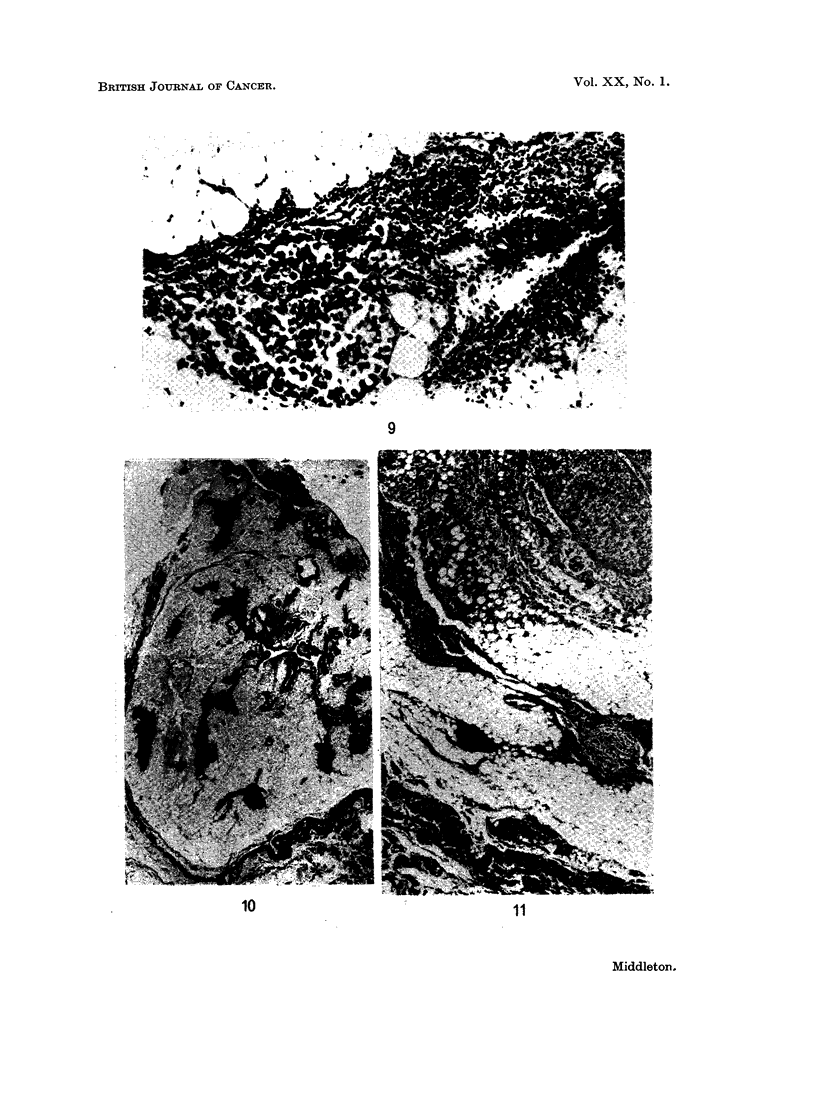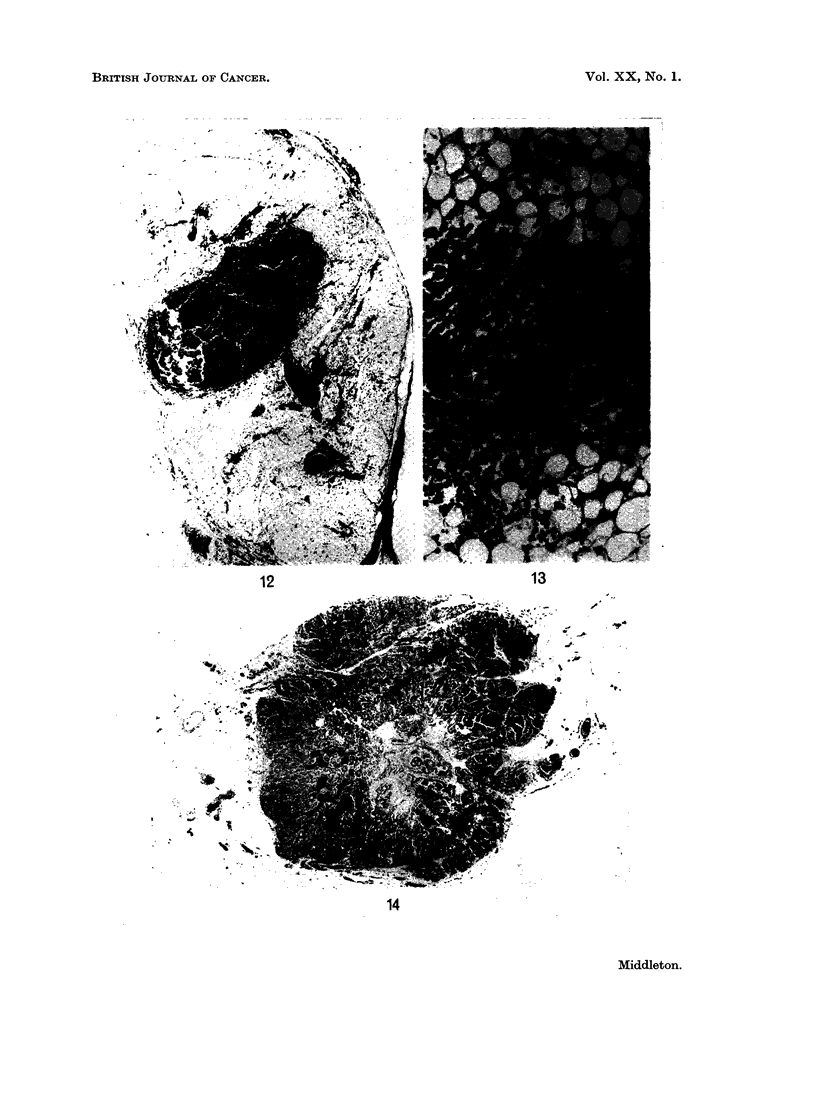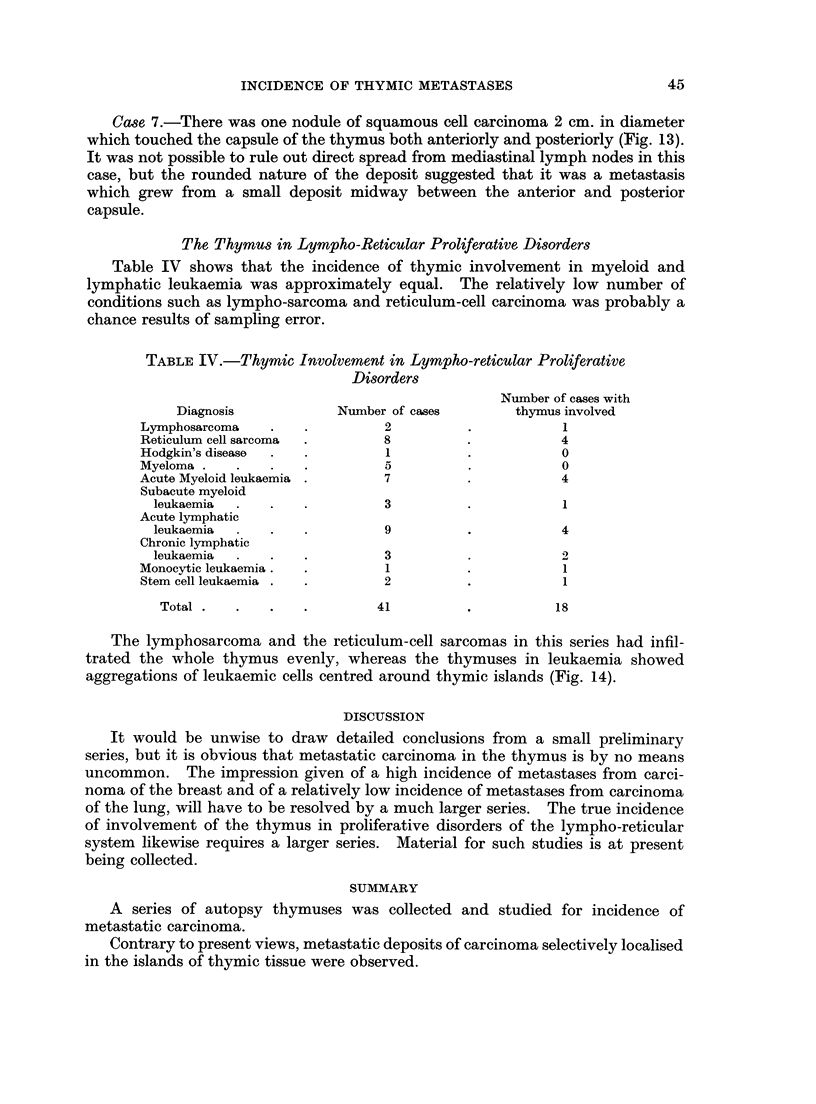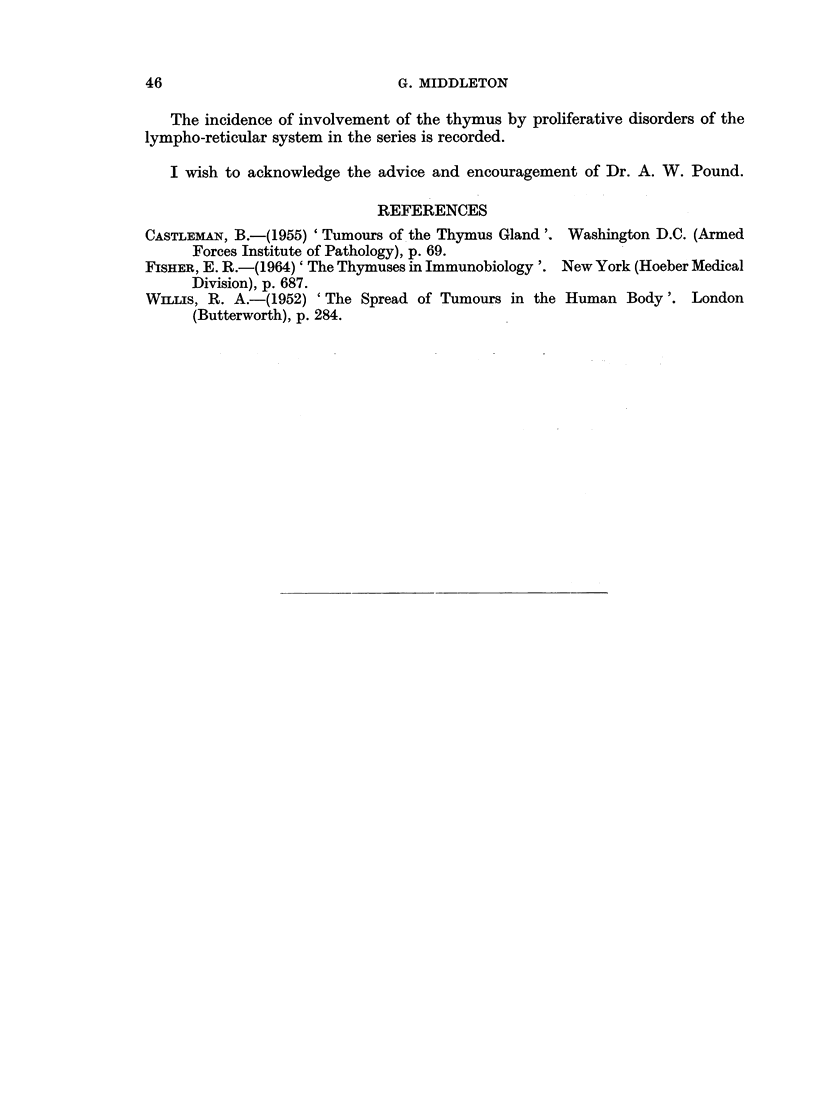# Involvement of the thymus by metastic neoplasms.

**DOI:** 10.1038/bjc.1966.6

**Published:** 1966-03

**Authors:** G. Middleton

## Abstract

**Images:**


### INVOLVEMENT OF THE THYMUS BY METASTATIC NEOPLASMS

G. MIDDLETON

*From the Department of Pathology, University of Queensland, Brisbane, Australia*

Received for publication November 2, 1965

IN his book, "The Spread of Tumours in the Human Body", Willis (1952) defined metastases as " growths which are separate from the primary growth and have arisen from detached transported fragments ", as distinct from direct spread. Reviewing the distribution of metastases from various sources of primary growth, he stated that he knew of no fully described case of genuine metastasis in the thymus. He quoted a case of his own with mammary carcinoma, where the thymus was involved by direct spread from the anterior mediastinal glands. Castleman (1955) made the following statement : " The most common cause of neoplastic involvement of the mediastinum is that of metastatic carcinoma. Although this usually means the mediastinal lymph nodes, the thymus gland is often obscured either by replacement of encroachment by the metastatic carcinoma ". However, he does not make it clear whether the thymus is involved by metastasis or direct spread. Fisher (1964) stated that little information concerning the thymus as a site for metastatic neoplasms had been recorded. He said that he had reviewed a limited number of cases of bronchogenic carcinoma without finding either macroscopic or microscopic metastases.

The purpose of this paper is to describe examples of metastatic carcinoma in the thymus, and to record the incidence of involvement of the thymus in proliferative disorders of the lympho-reticular system, which were discovered in a retrospective survey of autopsy material.

### MATERIAL AND METHODS

Thymuses were collected at autopsies done at the Brisbane Hospital, in the two-year period, January, 1963 to January, 1965. During this time 272 autopsies were carried out on patients who had benign tumours, malignant tumours, and proliferative disorders of the lympho-reticular system. Tissue was taken from the thymic region in 180 of these patients. Four blocks were cut from each sample of tissue. In 13 patients no thymic tissue was discovered in the original blocks, so further blocks were cut. No thymic tissue was found in the recuts. In the 167 patients remaining there were 171 examples of the disease entities under study. The discrepancy is explained by the fact that 4 patients had two conditions. It is probably best to consider the material as 171 " cases ", rather than 167 " patients ". Thus the patient who had both carcinoma of the stomach and malignant melanoma possessed the chance of thymic metastasis by both neoplasms, and for the purpose of tabulating incidence, is considered as two cases, rather than a single patient. Only one of the 4 had thymic involvement, which was leukaemic, in a patient with secondary adenocarcinoma in the lung.

### RESULTS

The categories in Table I in which there were no cases of thymic involvement, are not considered further. The incidence of metastases in the carcinomas is shown in Table II. There are no examples of involvement of the thymus by direct spread because these were excluded. It is seen from Table II, that the incidence of thymic metastases in 102 cases of carcinoma is approximately 7%. If the carcinomas which had not metastasized elsewhere are eliminated, the incidence becomes 10% of 74 cases. The low incidence of thymic metastases in carcinoma of the lung compared with the incidence in carcinoma of the breast is striking. The seven cases in which carcinomatous metastases were discovered in the thymus are considered in more detail in Table III. In Cases 1 and 2, the mediastinal lymph nodes were not commented on ; in Case 3, they were said not to be involved by carcinoma.

TABLE I.—*Incidence of Thymic Involvement in Cases with Various Types of Tumour*

| Group | Number of cases | Number of cases with thymic tissue in sections | Number of cases with thymic involvement |
|---|---|---|---|
| Carcinomas | 112 | 102 | 7 |
| Lympho-reticular | 44 | 41 | 18 |
| Benign | 11 | 11 | 0 |
| Cerebral | 13 | 13 | 0 |
| Sarcomas | 4 | 4 | 0 |
| Total | 184 | 171 | 25 |

TABLE II.—*Incidence of Metastases in the Thymus and Elsewhere in Cases with Carcinoma of Various Sites*

| Site of Primary Carcinoma | Number of cases | Number of cases with thymic metastases | Metastases elsewhere | | Metastases in Mediastinum | | |
|---|---|---|---|---|---|---|---|
| | | | Yes | No | Yes | No | Not known |
| Bile duct | 1 | nil | — | 1 | — | 1 | — |
| Bladder | 1 | nil | 1 | — | — | 1 | — |
| Breast | 10 | 4 | 10 | — | 6 | 1 | 3 |
| Cervix | 4 | nil | — | 4 | — | 4 | — |
| Large Bowel | 10 | nil | 6 | 4 | 1 | 9 | — |
| Larynx | 3 | 1 | 2 | 1 | 2 | 1 | — |
| Lung | 18 | 1 | 18 | — | 16 | 2 | — |
| Melanoma | 5 | nil | 5 | — | 2 | 2 | 1 |
| Ovary | 1 | nil | — | 1 | — | 1 | — |
| Pancreas | 5 | nil | 5 | — | 1 | 3 | 1 |
| Prostate | 14 | nil | 4 | 10 | 2 | 9 | 3 |
| Renal | 4 | nil | 4 | — | 0 | 3 | 1 |
| Stomach | 15 | 1 | 13 | 2 | 4 | 7 | 4 |
| Thyroid | 3 | nil | — | 3 | — | 3 | — |
| Trachea | 1 | nil | — | 1 | — | 1 | — |
| Uterus | 1 | nil | — | 1 | — | 1 | — |
| Unknown | 6 | nil | 6 | — | 3 | 3 | — |
| Total | 102 | 7 | 74 | 28 | 37 | 52 | 13 |

TABLE III.—*Details of Cases with Carcinomatous Metastases in the Thymus*

| Case no. | Sex | Age at Death | Primary Carcinoma | Clinical data | Organs other than thymus in which carcinoma was discovered at autopsy |
|---|---|---|---|---|---|
| 1 | F. | 53 | Breast | Left mastectomy and radiotherapy September, 1962, died March, 1963 | Right breast, cervical lymph nodes, pericardium, thyroid, perirenal connective tissue, cervical spine, calvarium. |
| 2 | F. | 62 | Breast | Left mastectomy 1942 ; right mastectomy 1944 ; excision of recurrent carcinoma in right mastectomy scar 1961 ; died May, 1964 | Skin of upper trunk, abdominal lymph nodes, lung, thyroid, liver, pancreas, kidneys, adrenals, pituitary. |
| 3 | F. | 44 | Breast | Radiotherapy for carcinoma of left breast which had involved left axilla November, 1963 ; died May, 1964 | Left axillary and left supraclavicular lymph nodes, spleen, lungs, adrenals, ovaries, uterus, brain, vertebrae and long bones. |
| 4 | F. | 53 | Breast | Right mastectomy October, 1964 ; died six weeks post operatively | Mediastinal, cervical and abdominal lymph nodes, spleen, lungs, heart, thyroid, liver, kidneys, adrenals. |
| 5 | M. | 43 | Stomach | Died three weeks after he presented with evidence of metastases | Abdominal and supraclavicular lymph nodes, pericardium, pancreas, adrenals, brain. |
| 6 | M. | 69 | Larynx | Presented as cervical lymph node metastases June, 1963 ; died June, 1964 | Mediastinal, cervical, supraclavicular and axillary lymph nodes, lungs. |
| 7 | M. | 69 | Lung | Died as a result of cardiac tamponade ; diagnosis not made in life | Mediastinal lymph nodes, pericardium. |

### *Histology of Thymuses with Carcinomatous Metastases*

*Case* 1.—Apart from the presence of metastases the thymic architecture was normal for the patient's age ; there were two distinct lobes, each with a fibrous capsule containing mainly fatty tissue with small islands of characteristic thymic tissue.

The deposits of carcinoma were distributed in such a way as to suggest true metastases rather than direct spread through the capsule (Fig. 1). The capsule was not involved by carcinoma in any of the sections. Most of the carcinomatous deposits were contiguous with thymic islands (Fig. 2). Tumour emboli could be seen in small vessels (Fig. 3). The tumour was poorly differentiated but was secreting mucus (Fig. 2).

*Case* 2.—In this patient the thymic islands were fewer than in Case 1. Most of the metastases were close to blood vessels with no adjacent thymic islands (Fig. 4 and 5), but there were some examples of metastases encroaching on thymic islands. The capsule was involved in several places where tumour cells could be seen in tissue spaces and in capsular lymphatics, but most of the deposits of carcinoma lay under uninvolved capsule (Fig. 4).

*Case* 3.—The metastases in this case were nearly all related to thymic islands. Many of them were expanding the islands from within (Fig. 6 and 7). No tumour emboli were observed in the vessels. The capsule was not involved in any of the sections.

*Case* 4.—In this case the effect of the metastases expanding the thymic islands was even more striking than in Case 3. Nearly all the thymic islands were involved in this way (Fig. 8 and 9). Tumour emboli were present in blood vessels. The capsule was involved in one small segment in one section only. This involvement was not contiguous with any of the metastases within the capsule.

*Case* 5.—In this patient, as in Case 2, the metastases were mainly in the perivascular connective tissue of medium-sized arteries and veins suggesting that they arrived via perivascular lymphatics (Fig. 10).

One metastasis surrounded a vein whose lumen was occluded by a thrombus containing malignant cells (Fig. 11). The capsule is not involved in any of the sections.

*Case* 6.—The sections showed two discrete nodules of squamous cell carcinoma neither of which was in direct contact with the capsule (Fig. 12). The capsule was not involved in the sections.

---

### EXPLANATION OF PLATES

FIG. 1 (Case 1).—Shows most of a transverse section of one lobe of thymus. The irregularly shaped darker areas are islands of thymic tissue and the lighter shaded nodules are metastases. The capsule is not involved by neoplasm. H. and E. ×7·5.

FIG. 2 (Case 1).—Interface between tumour above and thymic island below. The tumour is mucus secreting. Some of the epithelial cells in the thymic island have a squamoid appearance. H. and E. ×280.

FIG. 3 (Case 1).—Venules and lymphatics surrounding an artery are seen to contain tumour cells. Two thymic islands are seen, one top left, the other crossing bottom right diagonally. A segment of a metastasis is seen in the bottom right hand corner. H. and E. ×110.

FIG. 4 (Case 2).—Several vessels containing thrombus and malignant cells are shown. There is a metastasis adjacent to the artery to the right of centre of the field. H. and E. ×45.

FIG. 5 (Case 2).—Shows malignant cells in perivascular lymphatics and in the tissue spaces. H. and E. ×280.

FIG. 6 (Case 3).—Part of a transverse section of one lobe of thymus. The capsule is at the top. The elongated dark structures are thymic islands. The expansion (arrowed) occurring midway along one of these islands is due to a metastasis. H. and E. ×7·5.

FIG. 7 (Case 4).—Thymic island with malignant cells in the centre and thymic cells at the periphery. H. and E. ×280.

FIG. 8 (Case 4).—Transverse section of most of one lobe of the thymus. Almost all of the islands of thymic tissue are expanded by metastases. In the lower left of the field a dark line joining two paler rounded structures (arrowed) represents a narrow thymic island expanded at each end by a metastases. H. and E. ×7·5.

FIG. 9 (Case 4).—Shows a metastasis at the left hand end of a thymic island. H. and E. ×115.

FIG. 10 (Case 5).—Most of a transverse section of one lobe of the thymus is shown with its capsule around the periphery. Two pale areas (arrowed) adjacent to arteries are metastases. H. and E. ×6·5.

FIG. 11 (Case 5).—The thymic capsule is at the bottom of the field. A thymic island which crosses the field diagonally is being encroached upon at its upper end by a metastasis which surrounds the thrombosed vein seen in the upper right quadrant. H. and E. ×28.

FIG. 12 (Case 6).—Shows an ovoid deposit of squamous carcinoma which is separate from the thymic capsule on all sides. H. and E. ×7·5.

FIG. 13 (Case 7).—Shows a rounded deposit of carcinoma which is contiguous with the capsule of the thymus at the top of the field. At the bottom a thymic island is included in the fatty tissue which separates the deposit from the capsule. However, in another section, the deposit abuts against the capsule on both sides. H. and E. ×4·5.

FIG. 14.—Thymus from a female aged 60 years who died from acute myeloid leukaemia. The leukaemic cells appear to be " spilling out " from the thymic island into the surrounding fat. This appearance was present in all adults with leukaemic involvement of the thymus The thymuses of children with leukaemic involvement did not show this appearance as the interstitial tissue was much less bulky. H. and E. ×115.

1

2

3

4

Middleton

5

6

7

8

Middleton.

9

10

11

Middleton.

12

13

14

Middleton.

*Case* 7.—There was one nodule of squamous cell carcinoma 2 cm. in diameter which touched the capsule of the thymus both anteriorly and posteriorly (Fig. 13). It was not possible to rule out direct spread from mediastinal lymph nodes in this case, but the rounded nature of the deposit suggested that it was a metastasis which grew from a small deposit midway between the anterior and posterior capsule.

### *The Thymus in Lympho-Reticular Proliferative Disorders*

Table IV shows that the incidence of thymic involvement in myeloid and lymphatic leukaemia was approximately equal. The relatively low number of conditions such as lympho-sarcoma and reticulum-cell carcinoma was probably a chance results of sampling error.

TABLE IV.—*Thymic Involvement in Lympho-reticular Proliferative Disorders*

| Diagnosis | Number of cases | Number of cases with thymus involved |
|---|---|---|
| Lymphosarcoma | 2 | 1 |
| Reticulum cell sarcoma | 8 | 4 |
| Hodgkin's disease | 1 | 0 |
| Myeloma | 5 | 0 |
| Acute Myeloid leukaemia | 7 | 4 |
| Subacute myeloid leukaemia | 3 | 1 |
| Acute lymphatic leukaemia | 9 | 4 |
| Chronic lymphatic leukaemia | 3 | 2 |
| Monocytic leukaemia | 1 | 1 |
| Stem cell leukaemia | 2 | 1 |
| Total | 41 | 18 |

The lymphosarcoma and the reticulum-cell sarcomas in this series had infiltrated the whole thymus evenly, whereas the thymuses in leukaemia showed aggregations of leukaemic cells centred around thymic islands (Fig. 14).

### DISCUSSION

It would be unwise to draw detailed conclusions from a small preliminary series, but it is obvious that metastatic carcinoma in the thymus is by no means uncommon. The impression given of a high incidence of metastases from carcinoma of the breast and of a relatively low incidence of metastases from carcinoma of the lung, will have to be resolved by a much larger series. The true incidence of involvement of the thymus in proliferative disorders of the lympho-reticular system likewise requires a larger series. Material for such studies is at present being collected.

### SUMMARY

A series of autopsy thymuses was collected and studied for incidence of metastatic carcinoma.

Contrary to present views, metastatic deposits of carcinoma selectively localised in the islands of thymic tissue were observed.

The incidence of involvement of the thymus by proliferative disorders of the lympho-reticular system in the series is recorded.

I wish to acknowledge the advice and encouragement of Dr. A. W. Pound.

### REFERENCES

CASTLEMAN, B.—(1955) ' Tumours of the Thymus Gland '. Washington D.C. (Armed Forces Institute of Pathology), p. 69.

FISHER, E. R.—(1964) ' The Thymuses in Immunobiology '. New York (Hoeber Medical Division), p. 687.

WILLIS, R. A.—(1952) ' The Spread of Tumours in the Human Body '. London (Butterworth), p. 284.